# Supplementary material for: Are carbon emissions trading and green financial instruments synergistic? -Comprehensive quantitative research based on content analysis
Source: PLoS One. 2024 Mar 7;19(3):e0298601. doi: 10.1371/journal.pone.0298601 (PMC10919601; doi:10.1371/journal.pone.0298601)
Supplement: S1 Data — (ZIP) [file pone.0298601.s001.zip › Data/地级市Z_F工作报告.zip!/╦─┤¿/│╔╢╝╩╨/└┤╫╘╣1⁄2╓┌║┼í╛┬φ┐╦╩2╛▌═°í┐_2016.docx]

各位代表：

现在，我代表市人民政府向大会报告工作，请予审议，并请市政协委员和其他列席人员提出意见。

一、“十二五”及2015年工作回顾

“十二五”是成都发展历程中不平凡的五年，也是经济社会发展取得新成就的五年。五年来，我们深入贯彻党的十八大和十八届三中、四中、五中全会精神，认真落实习近平总书记系列重要讲话精神，在党中央国务院、省委省政府和市委的坚强领导下，在市人大市政协的监督支持下，按照中央“四个全面”战略布局和省委“三大发展战略”、市委“改革创新、转型升级”总体战略部署，主动适应经济发展新常态，积极应对各种困难挑战，完成了“十二五”规划主要目标任务，站上了“新常态、万亿级，再出发”的新起点，开创了成都科学发展的新局面。

——城市综合实力迈上新台阶。经济总量突破万亿元大关，人均地区生产总值突破1万美元。

——改革创新取得新进展。统筹城乡、行政审批、商事制度等重点领域改革深入推进，获批国家全面创新改革试验区、国家创新型城市试点和国家自主创新示范区，“创业之城、圆梦之都”“成都创业、创业都成”成为新的城市名片。

——城市国际影响力持续提升。与228个国家和地区建立经贸往来关系，落户世界500强企业268家，外国驻蓉领事机构15家。成功举办《财富》全球论坛、世界华商大会等国际盛会，被评为“全球最佳新兴商务城市”。

——市民生活不断改善。城镇和农村居民人均可支配收入年均分别增长11.2%、14.2%。新增就业超过166万人，基本实现人人享有社会保险。教育、文化、卫生等社会事业全面发展。连续四年蝉联“中国最具幸福感城市”榜首。

这五年，我们实施“交通先行”战略，构建现代立体交通体系取得重大进展。双流国际机场二跑道建成投用，成都天府国际机场获批立项。成兰、西成等9条铁路启动建设，成绵乐等3条铁路建成投运，成都东站、南站、铁路集装箱中心站全面建成，“蓉欧快铁”实现双向开行。第二绕城等4条高速公路竣工通车。开通运营2条地铁线。二环高架“两快两射”全面完成，初步形成“三环十六射”快速路网。

这五年，我们实施“产业升级”战略，经济发展质量稳步提升。形成电子信息、汽车、机械、食品4个千亿产业集群，成都高新区、经开区2个千亿园区，成为国家重要的高新技术产业基地、全国质量强市示范城市。全面完成“十二五”节能减排目标。现代服务业和都市现代农业加快发展，成为国家服务业综合改革试点城市、全国重要的优质农产品产区、西部重要的农产品加工中心和物流集散地。

这五年，我们实施“立城优城”战略，城市功能品质加快提升。天府新区获批国家级新区并加快建设。以“北改”为龙头的中心城区更新改造初见成效，曹家巷自主改造模式成为全国旧城改造样本。市政基础设施建设质量、运营标准和管理水平不断提升。出台环城生态区保护条例。以“四改六治理”为核心的城乡环境综合治理取得明显成效，获批国家生态文明先行示范区。城市总体规划获国务院批复。

这五年，我们实施“统筹城乡”战略，城乡面貌发生显著变化。财政投入“三农”超过1785亿元。城镇和农村居民人均可支配收入比由2.16∶1缩小到1.9∶1。市民“米袋子”“菜篮子”得到有效保障。统筹城乡综合配套改革9项成果在全国推广。获批全国第二批农村改革试验区，农村公共服务和社会管理设施标准化建设深入推进。探索形成“小组微生”新村建设模式。基本解决农村人口饮水安全问题。

这五年，我们实施“全域开放”战略，对外开放水平不断提升。实际利用外资、实际到位内资、外贸出口总额分别是“十一五”的2倍、2.5倍、3.3倍。国际（地区）航线达85条。成都关区实现通关一体化。中韩、中法等国际产业合作园落户，国际友好城市和友好合作关系城市达68个，与国内重点区域和城市合作加强，初步形成全方位、宽领域、多层次开放合作格局。

此外，全面完成“5？12”汶川特大地震灾后重建任务，有效应对了“4？20”芦山强烈地震，基本完成受灾乡镇重建和对口援建雅安任务。国家安全、国防动员、安全生产、机关事务、消防、人防、双拥工作切实加强，民族宗教、港澳台、工会、妇联、老龄、科协、慈善工作取得新进步，统计、档案、保密、气象、参事、地方志、哲学社会科学工作取得新成绩。

五年来，我们认真执行市人大及其常委会各项决议决定，自觉接受市人大法律监督和工作监督，主动接受市政协民主监督，办结人大代表、政协委员建议、提案6360件，满意和基本满意率99%以上。同时，全面加强政府自身建设，法治政府、服务型政府和廉洁政府建设取得新成绩，荣获中国法治政府奖，被评为中国服务型政府十佳城市。

需要特别说明的是，受国际国内经济形势、要素制约等多重因素影响，预计“十二五”地区生产总值、实际利用外资、进出口总额等指标增速放缓，与预期目标还有差距。但经过全市上下努力，“十二五”经济运行总体平稳、稳中有进、稳中有好，预计地区生产总值增速高于全国水平。

刚刚过去的2015年，面对国际环境错综复杂、国内经济下行压力加大的严峻形势，我们坚定信心、克难奋进、主动作为，经济社会保持平稳较快发展，预计实现地区生产总值10800亿元左右，增长8%左右；固定资产投资7006亿元，增长5.8%；一般公共预算收入1154.4亿元，增长12.6%；社会消费品零售总额4900亿元，增长10.5%；城镇和农村居民人均可支配收入分别增长8.5%、10%。重点做了七个方面工作：

（一）着力稳当前，确保经济发展稳中有进。坚持把稳当前作为当务之要，着力抓投资、促项目、保运行，及时出台了稳增长30条等系列政策措施，打出一套组合拳，确保经济健康发展。一是突出抓好招商引资。主动对接国家战略和规划，围绕城市发展需要和做大做强重点产业，针对重点地区、重点行业和重点企业，大力开展精准招商、专业招商和产业链招商，新签约引进重大项目520个，实际到位内资3540亿元、实际利用外资75亿美元，分别增长3.7%、5.6%。二是加快重大项目建设。强化资金、土地等要素保障，全力做好项目签约、落地、开工、建设、投产全程服务。落实领导干部联系重点项目制度，完善项目督促、督导、督查机制。实施重大项目837个，完成投资2450亿元。三是强化企业服务保障。加强企业融资、用能、运输等协调服务，及时解决企业实际困难。建立民营企业应急周转金，开展中小微企业壮大贷试点，中小微企业贷款余额增长11.3%。新增规模以上工业企业260家，产值过百亿工业企业增至10家，7家民营企业入选中国民营企业500强。

（二）着力谋长远，努力开创城市科学发展新局面。为实现“新常态、万亿级，再出发”起点上的新跨越、确保高标准全面建成小康社会，我们注重加强发展谋划和顶层设计。一是科学编制发展规划。着眼国际格局、国家战略、区域发展和成都实际，开展了“7+N”2025规划编制，并制定年度实施计划。编制完成“十三五”规划纲要，34个“十三五”重点专项规划正加快编制。二是优化城市发展空间。尊重城市发展规律，统筹谋划“双核共兴、一城多市”网络城市群大都市区发展格局。启动了大都市区规划编制，制定实施“独立成市”规划标准，推动二三圈层区（市）县“独立成市”形成都市新区。出台“小街区规制”规划技术管理规定，9个示范区已启动建设。三是加快建设对外大通道。新开通8条国际直飞客运航线，双流国际机场年旅客吞吐量突破4200万人次。成渝客专建成通车，实现高铁零的突破。成都至北京、西宁、昆明、格尔木等项目纳入国家中长期铁路网规划。启动实施“蓉欧+”战略，青白江铁路中心站提升为成都国际铁路港，成都至深圳、厦门、宁波、昆明“蓉欧+”货运班列开通运营，加快打造贯通欧亚、通江达海的“一带一路”大走廊。四是打造内陆开放新平台。中韩创新创业园落户并开展规划选址，中法生态园、中德创新产业园顺利推进，获批多式联运海关监管中心、铁路保税物流中心（B型），汽车整车进口口岸通过验收，建成进口肉类指定口岸，空港保税物流中心（B型）、成都公路口岸封关运行。新增世界500强企业6家、驻蓉领事机构3家，实现进出口总额410亿美元。五是加强多层次区域合作。制定了落实国家“一带一路”和长江经济带发展战略实施意见。强化与美国、墨西哥、古巴、巴基斯坦、印度等国家经贸往来，依托中欧区域合作政策试点加强与欧洲国家合作。推动构建成渝西昆贵“钻石经济圈”。与武汉、泸州签订港口物流合作协议，与宜宾、攀枝花、甘孜签订区域经济合作协议。

（三）着力调结构，提升经济发展质量和效益。坚持把调结构作为转方式的主攻方向，以调结构促转型、以促转型提质效，努力增强产业竞争力和国际话语权。一是推进工业加快转型升级。大力发展五大高端成长型产业，突出发展电子信息、汽车等重点优势产业，推动轨道交通、航空航天、软件制造、生物医药、新材料等产业加快发展。引进中国电子8.5代面板生产线、京东方柔性面板二期、中国电科网络信息安全等重大项目，东风神龙等项目加快建设，一汽丰田2.7L、成发航空发动机等项目竣工投产，汽车产量突破100万辆。攀成钢等50户企业淘汰落后产能。二是推动传统服务业高端化、新兴服务业项目化、服务业转型本土化。制定实施国际购物天堂五年行动计划，太古里等商业综合体建成开业。世界旅游目的地城市建设加快，万达文化旅游城等86个项目顺利推进。大力培育本土电商，推动本地产品上线销售，预计电子商务交易额6200亿元，增长20%。加快西部金融中心建设，实施经济证券化提升行动，新增A股上市公司10家、新三板挂牌企业69家，本外币存款余额突破3万亿元。成功申办G20财长和央行行长会议，成功举办2015世界机场城市大会等490个重大展会。三是推动农业组织化、市场化、标准化、品牌化。加强农业示范基地和农田水利建设，加快打造农业“天府品牌”，新增“三品一标”农产品37个。新增市级以上龙头企业、农民合作社、家庭农场611个，土地规模经营率58.6%。规划打造了26个赏花基地，休闲农业与乡村旅游收入达200亿元。全国转变农业发展方式现场会在成都召开并推广成都经验。

（四）着力添引擎，加快培育经济发展新动力。坚持抓创新就是抓发展、谋创新就是谋未来，把创新驱动发展作为面向未来的核心战略、作为推动城市转型的核心动力。一是启动全面创新改革试验。制定全面创新改革试验实施方案，以建设国家创新型城市、打造具有国际影响力的区域创新创业中心为目标，加快打通政产学研用协同创新和军民融合创新两个通道，扭住国家自主创新示范区、成都科学城、“创业天府”行动计划三大主要抓手，实施知识产权、人才发展、金融支撑、开放合作四大战略，强化政策、组织、法治、服务、舆论五大保障，系统推进全面创新改革试验。二是制定实施“创业天府”行动计划。搭建“菁蓉汇”平台，带动开展各类活动1000余场，吸引创客超过30万人次；成功举办成都全球创新创业交易会，达成全球创新创业“成都共识”。磨子桥（环川大）创新创业街区、电子科大“一校一带”、环交大智慧城等环高校成果转化区启动建设，“菁蓉镇”、蓉创广场等特色集聚区加快打造，新增孵化面积152万平方米。制定出台7大类46条政策，着力营造法治化、国际化、便利化的创新创业生态环境。被评为中国十大创业城市。三是规划建设成都科学城。秉持“创新为魂、科技立城”，在成都天府新区规划73平方公里建设成都科学城，布局一批国际领先研发机构和创新研发合作基地，加快聚集各类创新创业要素，打造西部创新第一城。加快天府创新中心等创新载体建设，引进中交建西南研发设计中心、清华能源互联网产业研究院、天府数智谷、诺基亚全球研发中心、中国电信天府云计算中心等重大创新项目。四是提升自主创新能力。全面启动国家自主创新示范区建设，加快推进科技成果“三权”改革，与北京大学、香港城市大学等高校共建产业技术研究院和科技园。强化“市域”服务理念，支持四川大学、电子科技大学、西南交通大学、西南财经大学等高校建设世界一流大学和一流学科。加快构建军民协同创新体系，商用无人机、喷气公务机等军民融合项目开工建设。新建研发机构80家，新增科技型企业超过1万家。

（五）着力打基础，不断提升城市功能品质。坚持城为民建、市为民享，努力做强城市基础功能、做美城乡环境、做优城市品质。一是强化重大基础设施建设。制定实施轨道交通加速成网计划，地铁1号线南延线开通运营、4号线试运行、3号线等7条线路加快建设、18号线开工建设，地铁在建里程237公里。沙西线建成通车，大件路外绕线基本建成。红星路等综合整治工程顺利推进。海绵城市和地下综合管廊建设开展试点。新建4G基站1.2万座，建成全国首个全光网城市。新投运110千伏以上变电站13座。李家岩水库获批立项。二是强化新区建设和旧城更新。天府新区“三纵一横”骨干道路投入使用，西部国际展览中心主体竣工，兴隆湖环境治理和锦江生态带综合整治顺利实施。“北改”工程实施重大项目69个，完成投资720亿元。“四改六治理”完成老旧院落改造944个、棚户区改造1.7万户、中小街道路面“黑化”维护120条。卫星城和区域中心城加快建设，街子、寿安、平乐、淮口等小城市和特色镇快速发展。三是强化城乡环境综合治理。加强城市科学化、法治化、标准化、精细化管理。开展了道路清洗除尘“本色行动”，推动建立城市垃圾分类处置体系试点。实施44条主干道提速和10个重要路口“短平快”改造，加强非机动车管理，公交机动化分担率提高2个百分点。新建城市公园绿地21万平方米，国家卫生城市通过复审。加强大气、水污染综合治理，强化农村面源污染防治和重点排污企业监控，基本完成水环境综合整治三年行动计划，PM10、PM2.5年均浓度分别下降13%、16.9%。

（六）着力促改革，切实增强经济社会发展活力。坚持积极稳妥、大胆创新，推出一批激活市场、释放活力、有利于经济社会协调发展的改革举措。一是加强行政体制改革。大力推进简政放权、放管结合、优化服务，调整取消行政许可项目28项，不再保留非行政许可类别，建设工程并联审批时限减少14个工作日。完成新一轮政府机构改革。积极打造B2G、R2G新型政企政民互动平台，努力营造零障碍、低成本、高效率政务环境。二是加快经济体制改革。深化商事制度改革，新登记各类市场主体24.9万户，增长33.7%，高出全国18个百分点。深化投融资体制改革，设立市级产业投资引导基金，推广“拨改投”“拨改租”模式，16个基础设施项目成功实施。加快国资国企改革。成为全国内贸流通体制改革发展综合试点城市。三是深化统筹城乡改革。深化综合改革示范片建设，建成123个新农村综合体。郫县、崇州、温江分别获全国农村集体经营性建设用地入市试点、土地经营权入股发展农业产业化经营试点、中小城市综合改革试点。成为国家新型城镇化综合试点、农村金融服务综合改革试点城市。

（七）着力惠民生，让城乡居民共享改革发展成果。坚持以人为本、民生优先，突出保基本、补短板、兜底线、促公平，持续增进民生福祉。一是完善就业社保体系。实施就业优先战略，支持高校毕业生、被征地农民、返乡农民工等重点群体就业创业，城镇新增就业26.4万人。实施城乡居民养老保险市级统筹，资助3.8万名残疾人参加养老保险，支出医疗救助金1.65亿元，为4.3万名困难家庭失能老人和80周岁以上老人提供居家养老服务。竣工保障性住房1.95万套。实施“百村万户”扶贫攻坚工程，全面加强甘孜州10个县对口帮扶。二是提升公共服务水平。优先发展教育事业，新增公办和公益性幼儿园学位2.5万个，投入18亿元实施义务教育免费、中职教育学费减免和帮困助学。县级公立医院和基层卫生综合改革深入推进，“医联体”试点顺利实施。完善公共文化服务体系，加强文化遗产保护和利用，成功举办第五届国际非物质文化遗产节。实现行政村（社区）健身设施全覆盖，成功举办全国第九届残运会暨第六届特奥会等大型体育赛事。三是深化平安成都建设。健全社会治安立体化防控体系，深入开展反恐防暴专项行动，严厉打击黄赌毒、非法集资、传销等违法犯罪行为。全面加强食品药品监管，全年未发生重大食品药品安全事故。强化特种设施、危化品、消防等领域安全监管和专项整治，生产经营性安全事故下降23.8%。全面完成“六五”普法。社会保持安定和谐。

市政府承诺的十大民生实事全面完成。

各位代表，这些成绩的取得来之不易，这是党中央国务院、省委省政府和市委正确领导的结果，是市人大、市政协和社会各界支持监督的结果，是全市人民共同努力的结果。在此，我代表市人民政府，向在各个领域和岗位上辛勤劳动、作出贡献的全市人民，向给予我们支持与监督的人大代表、政协委员、各民主党派、工商联和无党派人士、各人民团体、社会各界人士，向大力支持我们工作的中央和省驻蓉单位、解放军和武警驻蓉部队，向关心支持成都建设发展的港澳台同胞、海外华人华侨和国际友人，表示衷心的感谢并致以崇高的敬意！

在肯定成绩的同时，我们也清醒看到，我市经济社会发展还存在一些亟需解决的矛盾和问题：发展不够仍然是成都最大的实际，工业基础还不牢固，服务业需要提升发展，部分行业和企业生产经营困难，保持经济持续健康发展还需艰苦努力；经济增长动力转换尚未完成，转型升级任务仍然艰巨，市场主体活力有待进一步释放；交通拥堵、空气污染等“大城市病”亟待系统治理，城市建设管理任务依然繁重；城乡居民收入还不高，市民生产生活条件有待进一步改善；政府的行政能力和水平还需要进一步提升。对这些问题，我们将高度重视，认真加以解决。

二、“十三五”规划纲要说明及2016年工作建议

（一）“十三五”规划纲要说明

根据市委十二届六次全会《关于制定国民经济和社会发展第十三个五年规划的建议》，市政府组织编制了《成都市国民经济和社会发展第十三个五年规划纲要（草案）》，现提请大会审议。这里简要说明如下。

成都“十三五”时期经济社会发展的指导思想是：高举中国特色社会主义伟大旗帜，全面贯彻党的十八大和十八届三中、四中、五中全会精神，以马克思列宁主义、毛泽东思想、邓小平理论、“三个代表”重要思想、科学发展观为指导，深入贯彻习近平总书记系列重要讲话精神，全面落实中央“四个全面”战略布局和省委“三大发展战略”要求，坚持发展是第一要务，牢固树立创新、协调、绿色、开放、共享的发展理念，以提高发展质量和效益为中心，以抓好创新驱动、融入“一带一路”为主线，加快形成引领经济发展新常态的体制机制和发展方式，统筹推进经济建设、政治建设、文化建设、社会建设和生态文明建设，确保高标准全面建成小康社会，基本建成西部经济核心增长极，初步建成国际性区域中心城市，为建设国际化大都市、谱写伟大中国梦成都篇章奠定决定性基础。

成都“十三五”时期经济社会发展的具体目标是：

——建设西部经济中心。在提高发展平衡性、包容性、可持续性的基础上，保持经济中高速增长，推进产业向中高端发展。2020年地区生产总值达到2010年的三倍以上，城乡居民人均收入翻一番以上，城市综合实力显著增强。

——建设区域创新创业中心。国家创新型城市建设加快推进，创新驱动体制机制基本形成，创新创业能力显著增强，全面创新改革试验区建设任务全面完成，建成具有国际影响力的区域创新创业中心。

——建设国家门户城市。区域性国际综合交通枢纽功能日益完善。产业、企业、科技、人才、资本与国际市场深度融合，在全球创新链、产业链、供应链、价值链中的地位逐步提升，初步建成国家内陆开放型经济高地。

——建设美丽中国典范城市。初步构建生态制度体系、生态发展体系和绿色经济体系，优化提升城市形态、城市功能和城市品质，生态环境质量明显改善，生活家园更加美好，美丽成都建设取得明显成效。

——建设现代治理先进城市。法治城市建设深入开展。形成系统完备、科学规范、运行有效的制度体系，城市治理的科学化、法治化、现代化水平不断提升，初步建成治理体系和治理能力现代化先进城市。

——建设幸福城市。中国梦和社会主义核心价值观深入人心，公民素质和社会文明程度普遍提升。全市人民共建共享改革发展成果机制更加完善，人民生活水平和质量明显提高，人民群众的获得感、幸福感不断增强。

成都“十三五”时期经济社会发展的基本要求：一是坚持创新发展，培育经济发展新动力。二是坚持协调发展，形成均衡发展新格局。三是坚持绿色发展，建设生态文明新家园。四是坚持开放发展，开创合作共赢新局面。五是坚持共享发展，顺应人民生活新期待。

成都“十三五”时期经济社会发展的主要任务：一是加快创新驱动发展。二是加快建设开放门户。三是加快产业转型升级。四是加快市域空间转型升级。五是加快提升城市功能。六是加快信息化深度融合。七是推进生态文明建设。八是全面增进民生福祉。九是全力提升城市人文魅力。十是深入推进法治成都建设。十一是推动体制机制改革创新。此外，《纲要》还对规划实施提出了具体要求。

（二）2016年工作建议

2016年是我市实施“十三五”规划和7个2025规划的开局之年，也是推进结构性改革的攻坚之年。做好今年政府工作，要全面贯彻党的十八届五中全会精神和习近平总书记系列重要讲话精神，认真落实中央“五位一体”总体布局和“四个全面”战略布局，牢固树立和贯彻落实“五大发展理念”，深入实施省委“三大发展战略”和市委“改革创新、转型升级”总体战略，以提高发展质量和效益为中心，以抓好创新驱动、融入“一带一路”为主线，加强结构性改革，提高供给体系质量和效率，加快培育新的发展动能，整体提升社会生产力水平，促进发展成果共享，为建设国际化大都市奠定坚实基础。

2016年经济社会发展主要目标是：地区生产总值增长7%以上，固定资产投资总额7000亿元以上，一般公共预算收入增长7%，城镇和农村居民人均可支配收入分别增长7%、7.5%，城镇登记失业率控制在4%以内，万元地区生产总值能耗进一步降低。重点抓好九个方面的工作：

一是实施创新引领行动，加快建设国家创新型城市和具有国际影响力的区域创新创业中心。系统推进全面创新改革试验，实施创新型城市建设2025规划年度计划，提升全社会创新力、创业力和创造力，增强城市发展动力和核心竞争力。全社会研发投入增长15%，新增科技型企业1万家以上。

加快打通“两大通道”，激活创新创业资源。着力打通政产学研用协同创新通道，深入开展科技成果“三权”改革，提升环高校成果转化区水平，加快建设国家技术转移西南中心；协同在蓉高校共建世界一流大学和一流学科，联合建设新型产业技术研究院，争取设立西部国家科学中心；建立首台（套）奖励和保险补偿机制，支持企业组建研发机构。着力打通军民融合创新通道，搭建军民兼容国家重点实验室等协同创新平台，加快建设国家航空产业基地、信息安全等特色军民融合产业园，推动多领域深度融合发展。

扭住“三大主要抓手”，集聚创新创业要素。加快国家自主创新示范区建设，落实“6+4”先行先试政策，在科技成果转化、人才引进、科技金融结合等方面率先突破，打造制度创新和模式创新试验田。加快成都科学城建设，推进诺基亚全球技术中心、中国电信天府云计算中心等项目加快建设，引进一批技术转移、检验检测等服务机构。深入实施“创业天府”行动计划，提升“菁蓉汇”品牌和实效，形成全市创新创业整体效应；积极申办并办好中国？成都全球创新创业交易会；加强线上“创业天府”云孵化体系和线下创新要素大市场建设；大力发展各类众创空间，加强“菁蓉镇”、蓉创广场等创新创业集聚区建设。

营造国际一流创新创业生态环境，强化创新创业服务保障。实施知识产权、人才发展、金融支撑和开放合作战略。强化知识产权保护和运用，推动设立成都知识产权法院和成都知识产权交易所。制定具有比较优势的人才政策，吸引更多创客做安逸的“蓉漂”。设立新兴产业创业投资引导基金。加快构建以成都为核心的成德绵协同创新共同体，更大范围参与国际国内科技合作。强化政策、组织、法治、服务和舆论保障，加快建设创业之城、圆梦之都。

二是实施工业强基行动，加快建设国家先进制造业中心。实施成都制造2025规划年度计划，以大项目支撑大产业，以大平台承载大发展，加快培育新的经济增长点，筑牢城市发展产业基础。规模以上工业增加值增长6.5%。

夯实工业发展平台。加快“3+N”工业园区布局，启动高新区扩区工作，做强天府新区产业支撑，提升经开区工业承载能力，规划建设“大智造”“大创造”“大车都”板块，在中心城区以外的区（市）县优化形成一批20平方公里以上的工业集中发展区。加快国家临空经济示范区建设，规划建设成甘工业园，推动成雅、成资、成阿、成眉、成德工业园区加快发展。打造一批现代工业新区和智慧园区。

狠抓工业项目建设。开展工业“竞进拉练”，强化项目土地、资金、能源等服务保障。开工建设中国电科网络信息安全产业园、中国电子8.5代面板生产线等116个项目，加快建设中车成都产业园、京东方扩能、兵装集团汽车零部件产业园等150个项目，推动东风神龙等134个项目投产达产。完成技改投资1600亿元、工业投资2000亿元以上。

培育壮大产业集群。大力发展五大高端成长型产业，提高先进制造业、高新技术产业占工业的比重。做优做强电子信息产业，加快发展集成电路、智能终端、网络通信、光电显示、电子元器件等产业，打造世界软件名城。做大做强汽车产业，推动一汽大众、吉利沃尔沃等企业导入新车型，扩大产能、提升配套能力，力争引进更多整车制造企业。加快培育轨道交通、生物医药、精密机械、智能制造装备、新材料等千亿产业集群。大力推进质量强市。多措并举帮助企业降低生产和运行成本。新增规模以上工业企业200家以上。

三是实施服务业提升行动，加快建设国家服务业核心城市。实施服务业2025规划年度计划，提高生产性服务业占服务业的比重，增强城市集聚力、辐射力和影响力。社会消费品零售总额增长10%，服务业增加值增长8.5%。

加快西部金融中心建设。加强金融机构中心、交易中心和后台服务中心建设，支持本土金融机构发展，争取获批民营银行试点。推进移动金融、离岸金融创新和国家农村金融服务综合改革试点，扩大企业债券和公司债券发行规模，支持实体经济发展。完善金融要素市场功能，争取设立成都金融资产交易中心和成都股权众筹交易所。提升经济证券化水平和直接融资比重，新增A股上市公司10家、新三板挂牌企业50家。

加快打造国际购物天堂。推动传统商贸提档升级，促进国际商品保税展示中心、人南国际广场等项目加快建设。大力发展定制服务、体验式消费模式，扩大信息消费、健康养老等新兴消费。优化提升国际购物环境，实施境外旅客购物离境退税政策，增设国际货币汇兑便捷窗口，吸引更多国内外游客到成都购物，形成市民购、访客购、电商购多购并举消费局面。

加快建设世界旅游目的地城市。强化旅游标准化建设与管理，提升发展乡村旅游。加强重大旅游项目促建，推动万达文化旅游城等项目加快建设，启动凤凰山文化旅游主题公园、西部旅游总部基地等项目建设。推进青城山、花水湾？西岭雪山创建国家级旅游度假区，安仁古镇、平乐古镇？天台山景区创建国家5A级旅游景区，建立自驾游营地体系。加强国际旅游营销，打造旅游和美食标杆城市。旅游总人数2亿人次以上。

加快发展新兴先导型服务业。大力发展电子商务，积极引进跨境电商企业总部，加快培育本土电商，实施农村电子商务示范工程，推动更多“成都造”上线销售。积极发展现代物流，加快发展城市共同配送。西部国际博览城一期建成投用，办好G20财长和央行行长会议、世界航线大会等重大展会活动。培育发展检验检测认证产业。加快打造国际体育赛事名城，申办更多高级别国际体育赛事，办好国际男子职业网球协会125挑战赛、铁人三项世界杯赛等国际赛事。

四是实施融入“一带一路”行动，加快建设国家门户城市和内陆开放型经济高地。实施国际化城市建设2025规划年度计划，加强对外大通道建设，统筹推进高水平双向开放，拓展城市发展新空间，形成全方位开放新格局。

厚植对外交通发展优势。以打造国际枢纽空港城市为目标，启动成都天府国际机场及场外配套设施建设，实施双流国际机场优化提能工程，推动硬件改造、流程再造、服务提升，规划建设城市候机厅。以打造高铁枢纽城市为目标，加快西成客专、成贵客专和成兰、川藏、成昆铁路建设，加快成都北站扩能改造，力争成蒲铁路建成通车。以打造区域高速公路枢纽为目标，开工建设环成都经济区和新机场高速。

深入实施“蓉欧+”战略。以打造贯通欧亚、通江达海“一带一路”大走廊为目标，加快成都国际铁路港建设，拓展国际国内物流“两张网”，推动物流业与制造业、服务业“三业并举”，做强枢纽、节点、腹地、服务“四大支撑”，规划建设“蓉欧+”陆港产业园，加快打造国际口岸城市和国家向西向南开放桥头堡。“蓉欧快铁”国际端延伸至荷兰蒂尔堡等欧洲枢纽城市，国内端延伸至沿海沿边重要城市，全年开行300列以上。积极融入长江经济带建设，提升“成宜泸汉”港口联运水平。加快公路货运集散中心建设。开工建设一站式口岸综合服务中心。

做大国际合作平台。启动中韩创新创业园“一园一基地”建设，推动中法生态园、新川创新科技园加快建设，规划建设中德创新产业园、中古生物产业园，加快打造国际“五金小镇”。争取更多外国领馆和国际组织分支机构落户。实施外籍人士“家在成都”工程，为外籍人士在蓉工作生活创造更好环境。争取144小时过境免签政策。争创中国（成都）内陆自由贸易试验区。支持欧盟创新中心发展。

提升开放型经济水平。大力发展外向型经济，编制“一带一路”产业指导目录，强化维护权益、规避风险等政务服务，鼓励企业“走出去”开拓市场，参与“一带一路”沿线国家跨境园区和重大工程建设。建立政府一把手招商引资出勤率和成功率综合考核机制，持续提升专业化招商水平，引进一批核心竞争力强、带动作用大的项目。实际利用外资80亿美元以上，到位内资3600亿元以上。

增强区域合作整体效能。发挥“首位城市”作用，加强与德阳、绵阳、遂宁、资阳、眉山、乐山、雅安等成都经济区城市合作。推动与泸州、宜宾、甘孜、阿坝、凉山、攀枝花等地合作。加快成渝经济区一体化。加强成渝西昆贵“钻石经济圈”区域合作。深化与长三角、珠三角、京津冀等地区合作。

五是实施城建攻坚行动，加快建设宜业宜居城市。实施城建攻坚2025规划年度计划，统筹规划、建设、管理三大环节，地下地上并举，市区两级联动，整体提升城市承载能力和功能品质，让市民生活更方便、更舒心、更美好。

优化城市空间布局。以建设国际化大都市为目标，统筹生态、生产、生活三大布局，统筹空间、规模、产业三大结构，加快构建“双核共兴、一城多市”网络城市群大都市区发展格局，完成大都市区规划编制。加快二三圈层区（市）县“独立成市”形成都市新区，着力打造产城融合、职住平衡、均衡发展现代化新城区。大力提升中小城市和特色镇功能品质，持续推进“小组微生”幸福美丽新村建设。开展2049远景发展战略研究。

加强城市基础设施建设。提升成都天府新区产业承载能力和服务配套功能，启动高铁天府站建设。加强城市综合交通体系建设，加密市域公交线网，强化市域高速、快速路网络及重要转换节点建设改造，完成红星路、中环路、成洛路等道路综合改造工程，确保成温邛快速路建成通车，新改建村组道路1500公里以上。加强城区综合管廊建设，启动排水、排污“深隧”系统规划，建成成洛大道和成渝高速入城段综合管廊。推进国家级互联网骨干直联点带宽扩容。对接好国家重点项目工程包，争取更多国家专项建设基金。

大力实施轨道交通加速成网计划。以“中心城区加密成网、天府新区基本成网、轨道交通全域覆盖、实现轨道交通网络化”为目标，新开工地铁6号、8号、9号线等5个项目，加快5号、10号、18号线等9个项目建设，确保3号线、4号线一期开通运营，在建里程373公里，力争每年开通2条地铁，2020年运营里程达到500公里、在建里程150公里以上。启动都江堰有轨电车建设，加快IT大道、新津有轨电车示范线建设。启动市域铁路公交化运营改造。让市民出行更加便捷、准点、舒适。

提升中心城区功能品质。落实“小街区规制”技术管理规定，推动9个示范片区加快建设，加快构建城市主干道围合、中小街道分割、路网密度较高、公共服务就近配套的开放型街区，提升城市通透性、人情味和微循环能力。深入实施“北改”工程，加快天回镇、八里庄等重点片区改造，实施1.6万户棚户区、550个老旧院落改造，启动20个老旧市场调迁和太平寺机场搬迁，加快中心城区停车场建设。开工建设城市音乐厅，规划建设露天音乐广场及音乐坊、奥林匹克体育中心。

六是实施美丽成都行动，加快建设国家生态文明示范城市。牢固树立尊重自然、顺应自然、保护自然的生态文明理念，实施生态文明建设2025规划年度计划，推动城市科学发展、可持续发展，让成都的天更蓝、地更绿、水更清。

完善生态文明体制机制。加快构建生态文明制度体系，建立健全自然资源资产产权制度、国土空间开发保护制度、空间规划体系、资源总量管理和全面节约制度、资源有偿使用和生态补偿制度、环境治理体系、环境和生态保护市场体系、生态文明绩效评价考核和责任追究制度。加强资源集约节约高效利用，科学划定和严守生态红线，深化环评审批制度改革，大力推进建筑工业化和建筑垃圾资源化利用。

强化城乡生态建设保护。加强环城生态区建设和海绵城市建设，开工建设江安湖、北湖等5个水库，建成中和湿地等3个湿地公园。实施城市“增花添彩”工程，以再现“花重锦官城”胜景为目标，新增城市公园绿地10万平方米，新建街头小游园10个以上，加快26个赏花基地提档升级。实施龙泉山脉生态提升工程，筑牢龙门山、龙泉山“两山环抱”生态屏障。强化饮用水水源地保护,开工建设李家岩水库。启动成都大熊猫国家公园建设。积极申办第十二届中国园博会。

加强城乡环境综合治理。实施交通拥堵治理“九大行动”“九大计划”，系统治理交通拥堵问题。抓好大气环境综合治理，推动空气质量持续好转。实施水生态系统2025规划年度计划，推动生产、生活、生态用水“三水共治”。加强土壤污染和农村面源污染综合治理。强化城乡环境卫生管理、市容秩序管控和公共设施管护，开展城乡薄弱区域“脏乱差”治理攻坚行动，实现城区餐厨垃圾统一规范收运、集中无害化处理，确保城市干净、整洁、有序。

七是加强重点领域改革，激发经济社会发展新活力。充分发挥改革的突破性和先导性作用，扭住关键、精准发力，蹄疾步稳推进重点领域和关键环节改革。

深入推进统筹城乡改革。实施统筹城乡2025规划年度计划，稳步推进全国第二批农村改革试验区建设。以盘活农村土地、资本、劳动力资源为突破口，扎实开展土地承包经营权流转、继承和有偿退出试点，完善集体建设用地流转收益分配机制，稳妥推进郫县农村集体经营性建设用地入市试点，全面完成农村集体资产股份化改革试点，推动城乡生产要素平等交换、公共资源均衡配置。

加快转变农业发展方式。大力发展都市现代农业，加强农田水利基础设施和农业示范基地建设，加快打造创新高效、标准品牌、生态安全、开放合作、幸福共享五大新型现代农业。持续抓好“米袋子”“菜篮子”“果盘子”工程，启动100万亩菜粮基地提升行动计划，打造猕猴桃、茶叶、郫县豆瓣、邛崃基酒、油橄榄、彭州蔬菜等“天府品牌”。健全农业社会化服务体系，鼓励发展各类新型农业经营主体，扩大“农业共营制”试点。土地适度规模经营率达60%以上。农业增加值增长3%。

深化经济体制改革。探索建立以“管资本”为主的国有资产监管体制，加快国企整合和上市步伐，力争2—5家国企上市融资。营造宽松的市场经营和投资环境，鼓励、支持、引导非公有制经济发展。深化商事制度改革，全面实行全程电子化登记和电子营业执照应用。深化投融资体制改革，推广PPP模式，设立城市建设发展基金和新型城镇化基金，发行首批地铁“永续债”。深化国税地税征管体制改革。加强政府债务风险管控。完成内贸流通体制改革试点任务。

提升城市治理体系和治理能力现代化水平。强化依法治理，把法治思维和法治方式贯穿于城市治理全过程，推动政府依法行政、企业依法经营、市民依法行为。强化城市系统治理，统筹政府、社会、市民三大主体，发挥政府主导作用，推进社会组织依法发挥作用，增强市民参与城市治理的积极性和主动性，促进政府治理与社会自我调节、居民自治良性互动。强化城市数字治理，加强智慧城市建设，推动信息技术在政务服务、城市管理、民生服务等领域创新应用，促进城市治理更高效、营商环境更优化、市民生活更方便。

八是加强社会事业发展，让市民有更多获得感幸福感。围绕民生民向民怨，进一步实现好、维护好、发展好最广大市民的根本利益，让孩子们快乐成长、让青年人乐业创业、让老年人颐养天年，让市民生活更加幸福、更有尊严。

提升就业和社会保障水平。实施更加积极的就业政策，鼓励大学生、返乡农民工等重点群体以创业带动就业，实施技能成都计划，建立面向人人的就业服务体系，构建和谐劳动关系。建立更加公平可持续的社会保障制度，开展“全民参保登记计划”试点，有序推进机关事业单位养老保险改革，完善社会救助体系，保障困难群众基本生活。推行住房保障“租售补”并进模式，提高房地产市场管控水平，促进房地产业健康发展。启动新一批“百村万户”扶贫计划，做好甘孜州10个县对口帮扶。

增强城市文化软实力。积极培育和践行社会主义核心价值观。坚持教育事业优先，推动义务教育优质均衡发展，提升高中教育质量，鼓励社会力量兴办教育，加快发展职业教育，高水平办好成都大学，服务好在蓉高校发展，全面建成300所标准化公办幼儿园。大力发展文化事业，加强诚信文化和法治文化建设，深入开展文化惠民活动，弘扬川剧等地方优秀文化，加快发展文化创意产业。加强历史文化遗址和建筑保护，留住成都历史“根脉”和发展“年轮”。建成成都博物馆、杜甫千诗碑等项目，办好第七届中国博物馆（国际）博览会。

提高市民健康水平。深化医药卫生体制改革，加强医疗医保医药“三医”联动和“医联体”建设，强化医药分开和分级诊疗。推进中医药现代化。实施市民体质提升行动，大力开展运动成都、健康蓉城活动，办好第十三届市运会。完善以居家为基础、社区为依托、机构为补充的多层次养老服务体系，启动社区养老院三年达标计划，形成医养结合、全域覆盖的养老服务格局。着力优化人口结构，开展人口规划研究，落实“全面两孩”政策和居住证积分落户制度，吸引和留住更多年轻人及各类专业人才。

加强平安成都建设。高度重视公共安全，完善立体化社会治安防控体系，依法打击各类违法犯罪活动。加强金融风险预警监测，依法处置非法集资案件。加强大型群众性活动、轨道交通、危化品、特种设备等重点领域安全监管，实现电梯维护、保养、监管全覆盖。强化食品药品安全整治，创建全国食品安全城市。加强国防动员和后备力量建设。提高突发事件应急处置能力，确保城市安全、市民安宁、社会安定。

今年，我们将继续办好十大民生实事，包括城市改造、环境治理、扶贫解困、促进就业、教育助学、医疗卫生、社会保障、百姓安居、文化体育、基础设施等方面。

九是加强政府自身建设，加快建设法治政府、服务型政府、廉洁政府。坚持科学理政、团结共事，着力提升政府工作水平，打造讲政治、守规矩、有作为的公务员队伍，努力建设为民、务实、创新、清廉的人民满意政府。

加快建设法治政府。坚持依法行政，把政府工作全面纳入法治轨道，严格执行市人大及其常委会决议决定，自觉接受市人大及其常委会法律监督和工作监督，主动接受市政协民主监督，认真办理人大政协议案、提案和建议。依法全面履行政府职责，完善权力清单、责任清单和负面清单，切实做到法无授权不可为、法定职责必须为。广泛听取民主党派、工商联、无党派人士和工会、共青团、妇联等人民团体意见，高度重视社会监督和新闻舆论监督。

加快建设服务型政府。大力推进简政放权，进一步精简审批事项、简化审批流程、减少办事环节，加强事中事后监管，推进政务服务向基层延伸。加强新型智库建设，成立国际咨询顾问团，建立“市民参事”制度，提高政府决策科学化水平。加强创新型政府和学习型政府建设，强化公务员培训，完善B2G、R2G新型政企政民互动平台，拓展网上办事大厅功能，让企业和市民少走路、服务和信息多跑路。

加快建设廉洁政府。严格落实党风廉政建设各项规定，巩固党的群众路线教育实践活动、“三严三实”专题教育成果，持之以恒推进正风肃纪，切实防止“四风”问题回潮反弹。深入推进廉洁成都建设，健全惩治和预防腐败体系，加强行政权力监督制约，强化土地出让、工程建设等重点领域审计和监管。严格“三公”经费管理，完成机关公务用车改革，切实把更多财力用于保障和改善民生。

各位代表，让我们紧密团结在以习近平同志为总书记的党中央周围，在省委省政府和市委的坚强领导下，保持专注发展、转型发展的定力，团结一心、锐意进取，推动更高质量、更有效率、更加公平、更可持续的发展，为成都实现“十三五”发展总体目标、加快建设国际化大都市而努力奋斗！
